# Single-stage plasma-based correlated energy spread compensation for ultrahigh 6D brightness electron beams

**DOI:** 10.1038/ncomms15705

**Published:** 2017-06-05

**Authors:** G. G. Manahan, A. F. Habib, P. Scherkl, P. Delinikolas, A. Beaton, A. Knetsch, O. Karger, G. Wittig, T. Heinemann, Z. M. Sheng, J. R. Cary, D. L. Bruhwiler, J. B. Rosenzweig, B. Hidding

**Affiliations:** 1 Scottish Universities Physics Alliance, Department of Physics, University of Strathclyde, Glasgow G4 0NG, UK; 2 Cockcroft Institute, Sci-Tech Daresbury, Keckwick Lane, Daresbury, Cheshire WA4 4AD, UK; 3 Department of Experimental Physics, University of Hamburg, Hamburg 22761, Germany; 4 Deutsches Elektronen-Synchrotron DESY, Hamburg 22607, Germany; 5 Collaborative Innovation Center of IFSA (CICIFSA) & Laboratory for Laser Plasmas and School of Physics and Astronomy, Shanghai Jiao Tong University, Shanghai 200240, China; 6 Center for Integrated Plasma Studies, University of Colorado Boulder and Tech-X Corporation, Boulder, Colorado 80303, USA; 7 RadiaSoft LLC, Boulder, Colorado 80304, USA; 8 Particle Beam Physics Laboratory, Department of Physics and Astronomy, University of California, Los Angeles, California 90095, USA

## Abstract

Plasma photocathode wakefield acceleration combines energy gains of tens of GeV m^−1^ with generation of ultralow emittance electron bunches, and opens a path towards 5D-brightness orders of magnitude larger than state-of-the-art. This holds great promise for compact accelerator building blocks and advanced light sources. However, an intrinsic by-product of the enormous electric field gradients inherent to plasma accelerators is substantial correlated energy spread—an obstacle for key applications such as free-electron-lasers. Here we show that by releasing an additional tailored escort electron beam at a later phase of the acceleration, when the witness bunch is relativistically stable, the plasma wave can be locally overloaded without compromising the witness bunch normalized emittance. This reverses the effective accelerating gradient, and counter-rotates the accumulated negative longitudinal phase space chirp of the witness bunch. Thereby, the energy spread is reduced by an order of magnitude, thus enabling the production of ultrahigh 6D-brightness beams.

## Abstract (web summary)

Controlling and improving electron beam parameters are crucial for their application in free electron laser and X-ray sources. Here the authors generate quality electron beams with reduced energy spread from plasma accelerators by using a tailored escort electron bunch with the main accelerating bunch.

Acceleration of electrons in plasma waves harnesses electric fields on the order of tens of GV m^−1^ (refs 1, 2). This is orders of magnitude larger than in state-of-the-art radiofrequency-driven accelerator systems, and may allow to shrink even high-energy accelerators from km to metre-scale. Plasma accelerators have advanced from the generation of broadband electron distributions to point-like, quasi-monoenergetic electron beams^3^,^4^,^5^,^6^,^7^ in the last decade. The so-called Trojan Horse (TH) plasma wakefield acceleration^8^ and related schemes^9^,^10^,^11^,^12^ promise a further step change by decoupling the plasma wave excitation from the electron bunch generation in a highly flexible underdense plasma photocathode process. This allows generation and acceleration of transversally ultra-cold electron bunches with normalized transverse emittance down to *ɛ*_n_≈10^−9^ mrad. Reaching such low emittance has key significance for many of the most prominent accelerator applications, for example in high-energy colliders for high-energy physics, and for advanced light sources^13^. In high-energy physics experiments, emittance limits the focusability of particle beams and hence the obtainable luminosity, and in turn the event rate. This is why emittance damping rings are required to reach luminosity goals in colliders^14^. Obtaining nm-scale normalized emittances without the need for damping rings may therefore open up interesting avenues for high energy physics research. For similar reasons, electron emittance is crucial for light sources based on inverse Compton scattering, as the small spot sizes promote generation of large X-ray or γ-ray fluxes during the scattering process, as well as high-spectral brightness^15^,^16^, and for betatron or ion channel laser-based radiation sources^17^,^18^,^19^,^20^. Ultra-fast transmission electron spectroscopy is another important imaging technique at comparably low-electron energies on the few MeV scale, which likewise profits strongly from decreased transverse electron beam emittance, bunch duration and charge^21^. The electron beam 5D-brightness *B*_5D_≈2*I*_p_/(*ɛ*_*nx*_ *ɛ*_n*y*_), where *I*_p_ is the peak current and *ɛ*_n*x*,*y*_ are the normalized emittances in the transverse (*x*, *y*) directions, is a crucial figure of merit which amalgamates the bunch duration, charge and emittance. Emittance and brightness are key performance factors for free-electron lasers (FELs). Progress in this regard, particularly in the development of high brightness photocathode guns for radiofrequency-driven linear accelerators, had paved the way for the realization of research-enabling machines such as hard X-ray FELs^22^,^23^,^24^,^25^. The inherent ability of plasma accelerators to generate electron bunches with fs-scale durations and multi-kA currents^26^,^27^, enhanced by the TH strategy towards nm-scale transverse normalized emittance, suggests unprecedented 5D-brightness values approaching *B*_5D_≈10^20^ A m^−2^ rad^−2^ levels. This is order of magnitude brighter than state-of-the-art and may therefore have transformative impact, for example, for the realization of fifth generation light sources^28^.

However, the enormous accelerating fields in plasma accelerators inherently produce electron bunches with large correlated energy spread. In the strongly nonlinear blowout regime^29^ of plasma wakefield acceleration, the nearly sawtooth-shaped on-axis longitudinal electric field varies linearly with a steep slope along the co-moving coordinate *ξ*=*z−ct*, yielding acceleration gradients of the order of Δ*E*_*z*_∼0.3 MV μm^−1^ for a plasma wavelength of *λ*_p_≈100 μm, corresponding to a plasma electron density *n*_0_∼10^17^ cm^−3^. It results in a negative energy chirp: the energy distribution of the generated bunch is highly correlated to its longitudinal position inside the plasma wake, that is, the head of the bunch accumulates significantly lower energy than its tail during the acceleration process.

The accumulated correlated energy spread is problematic on a number of levels: first, extraction of the electron bunch from the plasma accelerator stage and transport^30^ can substantially deteriorate the beam emittance due to chromatic effects and non-matched transverse forces^31^,^32^,^33^. Similar effects occur during entry into a plasma stage, and are further multiplied when staged acceleration is required^34^. Second, the electron energy spread limits the realizability and performance of accelerator applications such as light sources. For instance, to allow proper micro-bunching and high-gain in FELs, the maximum relative energy spread Δ*W*_r.m.s._/*W*, should be less than the Pierce parameter^35^, *ρ*_FEL_ >Δ*W*_r.m.s._/*W*. Typical values for X-ray FEL's are in the range of *ρ*_FEL_ ∼10^−3^−10^−4^. Similarly, for example in case of inverse Compton scattering, low-electron beam emittance combined with small energy spread significantly narrows the on-axis photon radiation bandwidth^16^. While plasma accelerators produce bunches with comparably strong energy chirps, but inherently short bunch durations on the few-fs-scale, the situation is quite the opposite in state-of-the-art radiofrequency-based accelerators. Here, acceleration in the linac cavities typically leads to inherently monoenergetic bunches with very low correlated and uncorrelated energy spread, but in turn obtaining ultrashort electron bunches on the fs-scale requires magnetic compression techniques. In fact, energy chirps are purposefully generated in order to achieve sufficient compression in magnetic chicanes^36^,^37^. Various dechirping techniques are being developed and applied for state-of-the-art accelerators^38^,^39^,^40^ to subsequently improve the energy spread after compression. The electron beam 6D-brightness *B*_6D_≈*B*_5D_/0.1% Δ*W*_r.m.s._/*W* is used to quantify the combined current, transverse emittance and energy spread^25^,^41^.

Here, we show how to obtain electron bunches with ultrahigh 6D-brightness by controlled manipulation of the electron bunch longitudinal phase space inside a single-stage plasma accelerator.

## Results

### Loaded plasma wake in the 1D nonlinear regime

**Figure 1:**
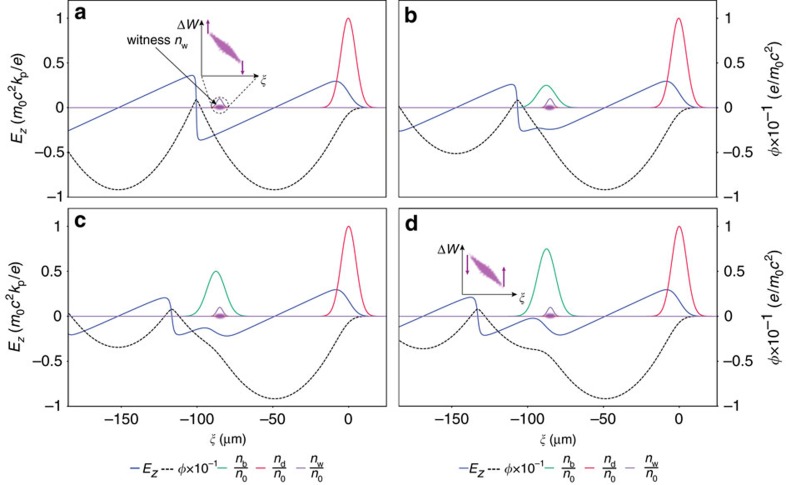
Beamloading of the plasma wake in 1D nonlinear regime. On-axis longitudinal electric field *E*_*z*_ (blue line) and electrostatic potential *φ* (dashed black line) in a plasma wave of density *n*_0_=1.1 × 10^17^ cm^−3^, driven by a non-evolving electron beam (red curve), propagating to the right. Adding an electron escort beam (green curve) with charge density *n*_b_ can load the wake and flatten or reverse the electric longitudinal field locally: (**a**) unloaded case (*n*_b_=0), where the position of the witness bunch *n*_w_ (purple curve) and its resulting energy chirp is indicated schematically, (**b**) *n*_b_/*n*_0_=0.5, (**c**) *n*_b_*/n*_0_=1.0 and (**d**) *n*_b_/*n*_0_=1.5. The results are obtained using the 1D nonlinear fluid model description. The electron witness bunch position and size (purple) is indicated, thus visualizing the electric accelerating field which would be sampled by the witness. The insets in (**a**,**d**) are the longitudinal phase spaces of the witness bunch, indicating the phase rotation for the (**a**) unloaded and (**d**) loaded cases.

A co-propagating Gaussian electron beam with peak density 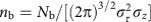, where *N*_b_ is the number of electrons and *σ*_r_, *σ*_*z*_ are the transverse and longitudinal r.m.s. beam sizes, respectively, can distort the accelerating plasma wakefield via beam loading^42^,^43^ if *n*_b_ becomes similar to the plasma electron density *n*_0_. Figure 1 visualizes this for the case of an electron beam driven plasma wakefield accelerator (PWFA) in the nonlinear 1D model^44^ (see Methods). Hence, the electron density ratio of *n*_b_*/n*_0_∼1 defines the threshold for overloading the wake. While the TH method allows to release low-emittance electron populations with charge tunable over many orders of magnitude, it is unable to reach the electron density levels required for beam loading without compromising the highest achievable brightness levels. This is due to strong space charge forces during the witness bunch formation, which increases the emittance. However, space charge forces decrease with *γ*^−2^ during acceleration, where *γ* is the relativistic Lorentz factor associated with the accelerating electron beam. To exploit this, an additional electron escort population is released once the witness bunch is relativistically stable, such that the escort overlaps with the witness bunch during the trapping process, but without destroying its emittance. The escort electron population is tuned to a much higher charge values than the witness bunch, for example, by increasing the corresponding photocathode laser intensity and/or its Rayleigh length. The amount of released charge can thus be regulated to flatten the local field (Fig. 1b), or can be further increased to levels such that the escort bunch overloads the wake and thus reverses the slope of *E*_*z*_ locally for the remainder of the acceleration process, as shown in Fig. 1c,d. This allows adjustment of the witness energy chirp by counter-rotation of the longitudinal phase space to arbitrary positive or negative values, or—most important—to minimize it.

### Particle-in-cell simulation of the dechirping technique

**Figure 2:**
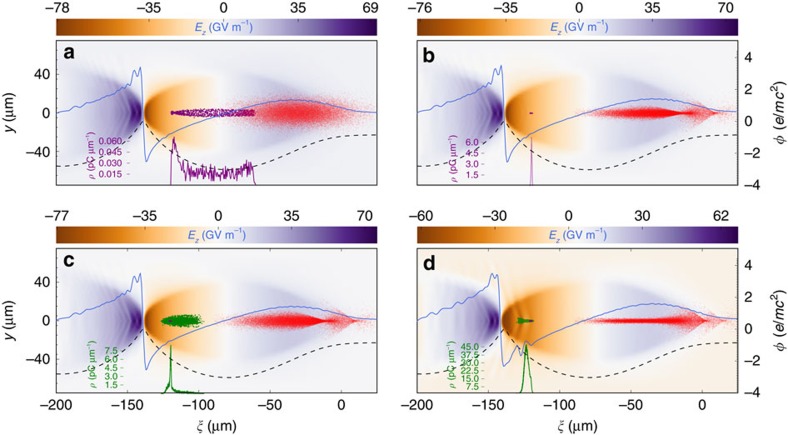
Numerical simulation of the dechirping process. Three-dimensional particle-in-cell simulation results with VSim^63^, showing snapshots of the driver beam (red) and the electric field *E*_*z*_ with on-axis lineout (blue solid line), electrostatic on-axis potential *φ* (black dashed line), and witness (purple) and escort bunch (green) longitudinal charge profiles. In (**a**) at *z*_acc_=0.075 cm, the witness bunch is just being released by the laser pulse ionization front (not shown), in (**b**) at *z*_acc_=1.6 cm, the witness is fully formed and accelerated to *W*≈500 MeV. In (**c**) at *z*_acc_=1.65 cm, the escort bunch is released and begins to overload the wakefield, and in (**d**) at *z*_acc_=2.4 cm, the escort bunch is fully trapped, overlaps with the witness bunch and has reversed the local accelerating field slope Δ*E*_*z*_/Δ*z*.

To verify the concept, we performed 3D particle-in-cell simulations (see Methods for details). As shown in Fig. 2a,b, a high 5D-brightness witness bunch (purple) is initially produced in a strong blowout with a longitudinal electric wakefield maximum of *E*_*z*,peak_≈80 GV m^−1^ based on pre-ionized lithium plasma (*λ*_p_≈100 μm), driven by a FACET-II (ref. 45) scale electron bunch (parameters are summarized in Supplementary Table 1). The TH witness bunch release laser pulse with normalized intensity *a*_0,w_=0.1, focal spot size *w*_*o*,w_=7 μm and duration *τ*_w_=25 fs generates the witness (purple) with charge *Q*_w_≈5 pC at the beginning of the plasma stage (Fig. 2a). The snapshot in Fig. 2a visualizes the trapping process with the transient longitudinal charge density *ρ*(*ξ*) plotted as inset, just before trapping compresses the witness bunch to a length of *σ*_w,z_≈2 μm, corresponding to a peak current of *I*_p_≈2.0 kA. At a normalized emittance of *ɛ*_n*x*,*y*_≈3.6 × 10^−8^ mrad, this corresponds to a 5D-brightness of *B*_5D_≈3.0 × 10^18^ A m^−2^ rad^−2^.

When this bunch reaches the energy of *W*≈500 MeV after an acceleration length of *z*_acc_≈1.6 cm, it is relativistically stable and much more immune to space charge forces when compared with the initial bunch during its trapping and formation process (Fig. 2b). Now, the second, more intense, softer focused and longer laser pulse (*a*_0,e_=0.11, *w*_0,e_=10 μm, *τ*_e_=80 fs) releases the escort beam with a charge of *Q*_e_≈184 pC—more than one order of magnitude compared to the witness bunch. Same holds for escort charge density *ρ*(*ξ*) (green) and the corresponding peak current. Trapping of this population in the accelerating phase of the wakefield strongly overloads the wake and reverses the slope of *E*_*z*_ (Fig. 2c,d) locally. The average ionization front position of the laser pulse which releases the escort bunch sweeps over the release position *ξ* of the witness bunch in the co-moving frame of the wake. Reflecting the intensity dynamics of a co-propagating focusing Gaussian Ti:Sapphire laser pulse with central laser pulse wavelength of *λ*_L_≈800 nm, the ionization front first starts to appear at the center of the 80 fs Gaussian laser pulse, where the tunnel ionization threshold is reached first. For the used peak intensity value, this happens before the geometric focus in the laboratory frame is reached. During further focusing, the ionization front then moves forward with respect to the Gaussian temporal intensity profile of the laser pulse, which in the co-moving frame means to higher values of *ξ* with respect to the plasma wake field. It reaches maximum *ξ* at the geometric focus position of the laser pulse, and then moves back towards the laser pulse intensity center as the pulse diffracts^46^,^47^. This single-cycle ionization front phase oscillation is larger in amplitude Δ*ξ*, the larger the peak intensity and the larger the Rayleigh length, is also dependent on the laser pulse duration and defines a maximum length of the produced electron bunch and its charge. While for the escort beam, this oscillation amplitude should be large, it should be small for the witness bunch. In case of the witness bunch, this also allows for an estimation of the witness residual energy spread, which will be discussed later. The optimization requirements for the witness bunch (low emittance, small energy spread and high current) and for the escort bunch (high charge, much longer duration than the witness) are therefore substantially different. Fortunately, the inherent flexibility of the scheme allows to tune both bunches independently, and to choose the parameters of the injection lasers in a wide range so that different regimes of ionization front movement can be accessed. The much longer escort Rayleigh length *Z*_R,e_=π*w*_e_^2^/*λ*_L_≈392 μm when compared with the witness *Z*_R,w_=π*w*_w_^2^/*λ*_L_≈192 μm, the significantly larger dimensionless light amplitude *a*_0,e_=0.11>0.1=*a*_0,w_ and the longer pulse duration *τ*_e_=80 fs >*τ*_w_=25 fs in combination lead to a desired large ionization front phase oscillation amplitude of approximately Δ*ξ*≈5 μm as observed in the simulation in case of the escort bunch production, while the ionization front is quasistatic in case of the witness bunch production. The accumulating beam-loading during trapping of the high-charge escort bunch production increasingly distorts not only the electric fields, but also the underlying electrostatic potential *φ*, as shown in Fig. 1 (dashed black line), and leads to significant elongation of the plasma blowout, as seen in Figs 1 and 2c,d. At the bottom line, the region where the escort bunch overloads the wake—the dechirping region—is much longer than the witness bunch, which can therefore be fully dechirped from head to tail.

**Figure 3:**
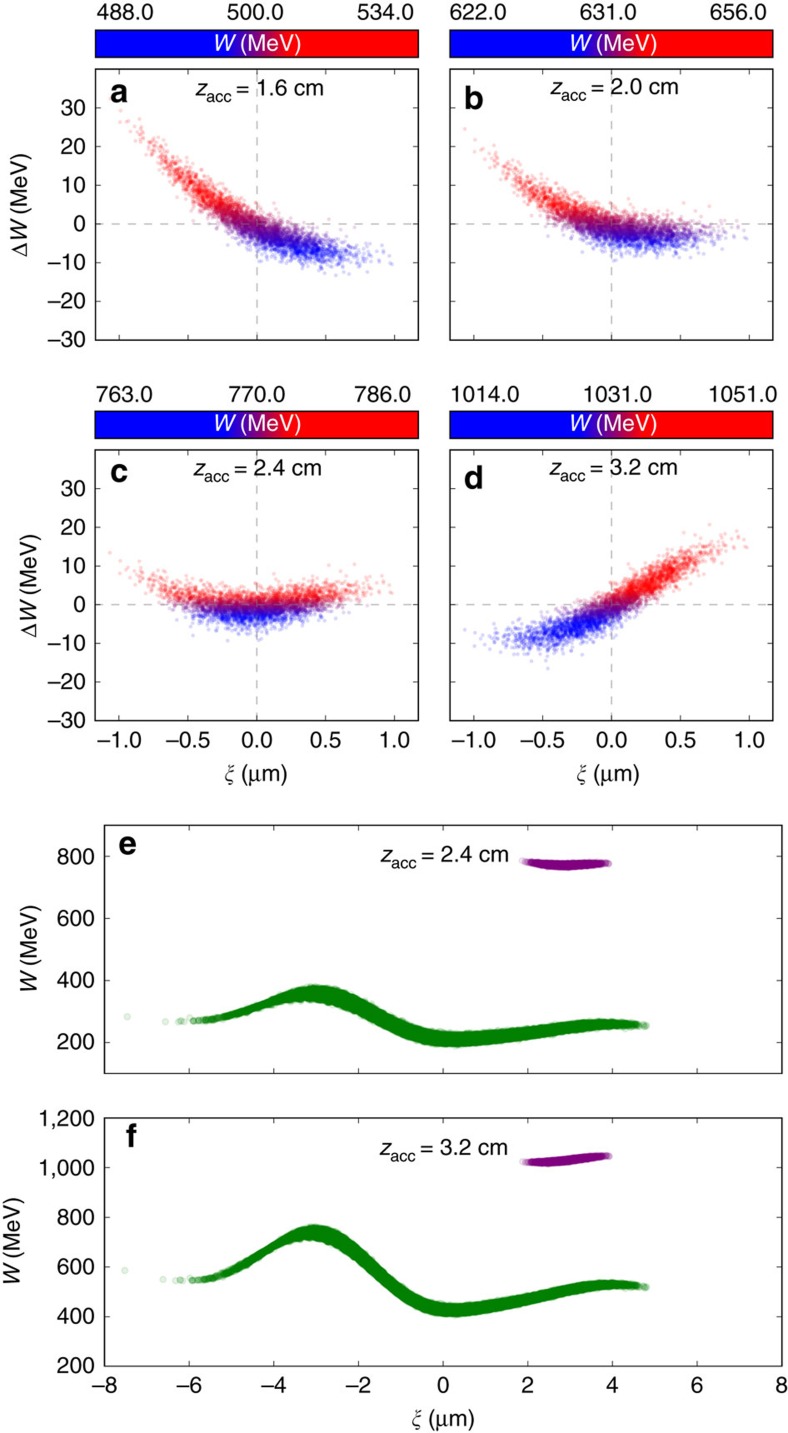
Witness bunch longitudinal phase space. 3D VSim results showing the longitudinal phase space evolution of the witness bunch just before release of the escort bunch (**a**) after Δ*z*_acc_≈0.35 cm of dechirping (**b**) and at *z*_acc_≈2.4 cm at optimum dechirping (**c**). Further counter-clockwise phase-space rotation generates bunches with positive energy chirps (**d**). (**e**,**f**) show the fully dechirped case and the overchirped case, respectively, where the witness (purple) and escort (green) beam are clearly separated by energy.

Figure 3 summarizes the corresponding longitudinal phase space evolution associated with the simulation discussed in Fig. 2. Immediately before the escort bunch is generated, the witness bunch exhibits the typical negative chirp, shown in Fig. 3a. This situation corresponds to Fig. 1a (unloaded wake) and Fig. 2b. Once the escort is produced and trapped at around the same position *ξ* as the previously produced and accelerated witness bunch, however, the locally reversed *E*_*z*_ begins to rotate the longitudinal phase space of the witness bunch counter-clockwise, while the witness bunch in total is further accelerated at slightly reduced level. Consequently, the witness bunch absolute energy spread is reduced strongly and as acceleration progresses the correlated energy spread reduces, reaching a minimum of Δ*W*_r.m.s._≈2.56 MeV at a mean energy of 770 MeV after *z*_acc_≈2.4 cm (Fig. 3c). This would be an optimal extraction point for key applications which require minimized energy spread. It is also possible to further rotate the phase space in order to generate positive energy chirps, which corresponds to the situation depicted in Fig. 3d. As shown in Fig. 3e,f, although necessarily spatially overlapping, the witness and escort electron population are at all times strongly separated in energy, which allows straightforward separation.

**Figure 4:**
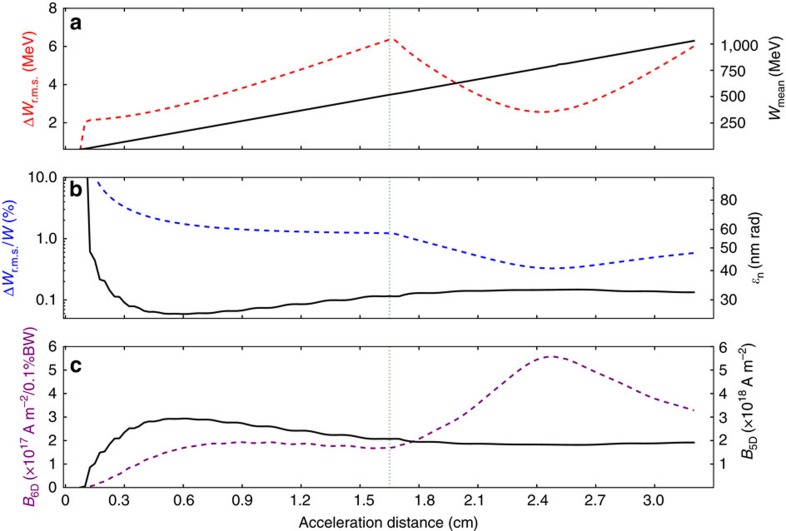
Evolution of the witness bunch parameters. (**a**) r.m.s. energy spread Δ*W*_r.m.s._ and central energy *W*_mean_; (**b**) relative energy spread Δ*W*_r.m.s._/*W* and normalized transverse emittance *ɛ*_n_; and (**c**) 5D- and 6D-brightness. At *z*_acc_≈2.4 cm in the lab frame, the optimum dechirping point and hence the maximum 6D-brightness is reached.

The results of these proof-of-concept simulations for the witness bunch parameters are summarized in Fig. 4. While the mean witness energy *W* increases linearly (black solid line, right *y*-axis), the absolute energy spread Δ*W*_r.m.s._ (red dashed line, left *y*-axis) increases significantly up to the escort release point at *z*_acc_≈1.6 cm, as expected (Fig. 4a). The Δ*E*_*z*_ reversal, generated by the escort bunch, then subsequently reduces the accumulated energy chirp until the minimum energy spread is reached at *z*_acc_≈2.4 cm. The active dechirping method includes head and tail of the witness bunch, which increases its efficiency. The minimum total energy spread is approximately the same as at the very beginning of the witness bunch acceleration process at *z*_acc_≈0.15 cm. The relative energy spread Δ*W*_r.m.s._/*W* (blue dashed line, left *y*-axis—note the log scale) reduces from nearly 2% before dechirping sets in, down to a minimum of ∼0.3% at a mean energy of *W*_mean_≈774 MeV, as seen in Fig. 4b. After the compensation of the correlated energy spread, the absolute energy spread Δ*W*_r.m.s._ rises again due to continuing phase space rotation, which leads to overcompensation. The witness bunch normalized transverse emittance *ɛ*_n_ (black solid line, right *y*-axis), is almost unaffected by the escort bunch and continues to stay at tens of nm-rad levels during the longitudinal phase space rotation. Finally, in Fig. 4c the 5D and 6D-brightness evolutions are plotted. The 6D-brightness reaches record values of *B*_6D_≈5.5 × 10^17^ Am^−2^ rad^−2^/0.1% Δ*W*_r.m.s._/*W*. Supplementary Movie 1 (with description provided in Supplementary Fig. 1 and Supplementary Note 1) visualizes the concept and the witness parameter evolution. It should be noted that the obtained values are significantly limited by the computational load of the 3D-simulations. At larger plasma wavelengths (thus simulation boxes), with other plasma species, and fully resolved laser pulses, the energy spreads, emittance and brightness values have the potential to be further improved by at least one order of magnitude.

### Calculation of the residual energy spread

**Figure 5:**
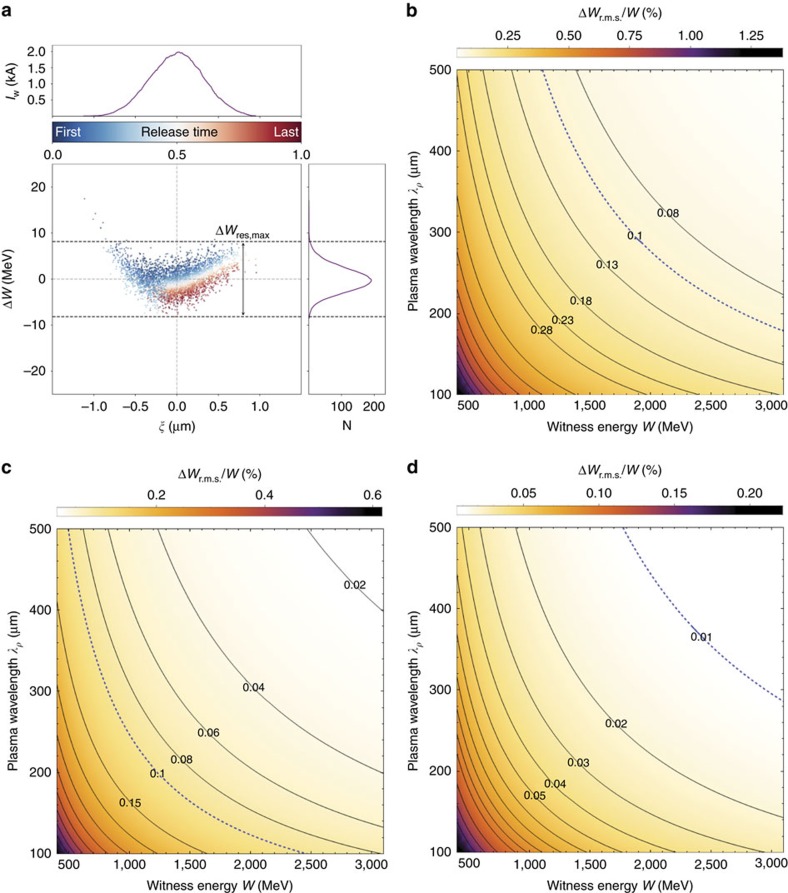
Residual energy spread from simulation and scaling prediction. Absolute residual witness energy spread from simulation (**a**) and relative energy spread from scaling predictions (**b**–**d**). In (**a**), the residual energy spread as observed in the PIC-simulation at minimum energy spread position (corresponding to the case depicted in Fig. 3c) is shown with color-coding of laser-released witness electrons, where position 0 indicates the first electron released and position 1 indicates the last electron released. Electrons which are released earlier (blue) are gaining more energy than those released later (red), in accordance with the numerical scaling estimation. **b**–**d** show relative energy spread predictions based on the residual energy spread scaling for a witness release laser spot size of *w*_0,w_=10 μm (**b**), *w*_0,w_=7 μm (**c**) and *w*_0,w_=4 μm (**d**) in dependence of witness bunch energy and wavelength of the accelerating plasma wave. Isolines depict the combinations of constant relative energy spread for selected values.

A fully chirp-compensated bunch or other longitudinal phase space shapes can be produced at arbitrary mean energy, as one can either vary the escort charge, its release position and/or the plasma stage length. The inherent residual energy spread of the witness may be approximated based on the different release times of individual witness electrons by the co-propagating laser pulse as Δ*W*_res,r.m.s._≈2π/5 *E*_*z*,trap_ *w*_0,w_^2^/*λ*_L_ (see Methods). Figure 5a visualizes the dechirped bunch of Fig. 3c with a color-coding of the release times, revealing that the electrons which were released first (blue) are accelerated longer and hence gain more energy than electrons born last (red). Using the witness bunch release laser parameters of *w*_0,w_=7 μm, *λ*_L_=800 nm, and an electric accelerating field at trapping position of *E*_*z*,trap_=33.2 GV m^−1^ as seen in the simulation, the scaling predicts a Δ*W*_res,r.m.s._≈2.55 MeV. This is in excellent agreement with the minimum energy spread of Δ*W*_res,r.m.s._≈2.56 MeV as retrieved from the simulation.

A further simulation was performed at reduced resolution to demonstrate the scalability of the concept to higher electron energies numerically (Supplementary Fig. 2 and Supplementary Note 2). Here, the relative energy spread amounts to Δ*W*_res,r.m.s._/*W*_mean_≈0.15% at mean energy of *W*_mean_≈1.6 GeV, and the corresponding slice energy spread is at the 0.1% level. This is likewise in excellent agreement with the residual energy spread scaling. The experimental implications are that strong focusing and operation at longer plasma wavelengths can decrease the residual energy spread. Figure 5b–d shows predictions of a generalized scaling which uses the linear wave-breaking limit as approximation for the electric field accelerating the witness (see Methods). The experimentally relevant relative energy spread, which naturally decreases as ∝ 1/*W*_mean_ on top of a minimized residual energy spread Δ*W*_res,r.m.s._, is plotted for different values of plasma wavelengths and laser focus sizes *w*_0_. For example, the scaling suggests that at *λ*_p_=500 μm and *w*_0,w_=7 μm, a relative energy spread of Δ*W*_res,r.m.s._/*W*_mean_≈0.03% can be obtained at an electron energy of *W*_mean_≈2 GeV (see Fig. 5c), and that for *w*_0,w_=4 μm, plasma wavelengths of a few hundred μm at energies around 2 GeV and beyond, the relative energy spread can reach values of Δ*W*_res,r.m.s._/*W*_mean_ <0.01%.

## Discussion

A tuneable and flexible scheme for minimization of energy spreads in plasma wakefield accelerators by approximately an order of magnitude is presented. The energy compensation occurs in a single stage at constant plasma density profile—the same stage where witness bunch generation and acceleration take place. This is a far-reaching advantage. First, otherwise extraction of energy-chirped witness bunches from plasma accelerator stages and their transport is highly problematic, and second the minimized energy spread enables a strongly broadened range of applications. While the technique is in principle applicable also for purely (for example, two-color) laser driven schemes, the dephasing-free PWFA with up to metre-scale acceleration in a single-stage and multi- or even tens of GeV energy gains, is of particular interest. The highest impact may be expected from applying the scheme to witness bunches with ultrahigh 5D-brightness, as accessible by the underdense photocathode Trojan Horse scheme. In this case, reduced energy spread is combined with ultrahigh 5D-brightness, leading to unprecedented 6D-brightness values. Experimentally, the production of the escort bunch for chirp control adds no additional qualitative challenges compared to the TH scheme, for which first signatures have recently been observed at SLAC FACET, the Facility for Advanced Accelerator Experimental Tests with its pioneering electron beam driven plasma wakefield accelerator program^48^. The concept is scalable towards higher energies and can remove correlated energy spread nearly completely, just leaving the minimized residual uncorrelated energy spread. Particular impact of the approach may be expected for light source applications. For example, in inverse Compton scattering setups higher *γ*-photon beams can be obtained with decreased spectral bandwidth if the electron energy spread can be minimized, or hard X-ray FEL's could be realized experimentally already at few GeV electron energies. The combination of low emittance, ultrahigh brightness and low energy spread may allow to beat the Pellegrini criterion^49^ and the Pierce parameter *ρ*_FEL_ at the same time, and to achieve ultrahigh gain. The Pellegrini criterion *ɛ*_n_≤*γλ*_r_/4π states that the transverse normalized emittance *ɛ*_n_ of the electron beam must be smaller than the obtainable diffraction limited FEL photon beam wavelength *λ*_r_, which is clearly satisfied at emittance levels of *ɛ*_n_ ≈10^−8^ mrad in principle even for sub-angstrom hard X-ray FELs. The derived residual energy spread scaling together with scalability to higher energies indicates that relative energy spread levels Δ*W*_r.m.s._ /*W*≈0.01% can be reached at few GeV energy levels, which would be an order of magnitude better than the threshold to satisfy Pierce parameter levels of *ρ*_FEL_ <0.1% as required for a hard X-ray FEL. Electron energies of few GeV can nowadays be reached straightforwardly in a single plasma stage. For example, an electron beam at energy *W*=3 GeV and with a normalized emittance of *ɛ*_n_=40 nm and a peak current of *I*_p_≈2.0 kA, dechirped to relative energy spreads of Δ*W*_r.m.s._/*W*≈0.085% (corresponding to a residual energy spread of Δ*W*_res,r.m.s._≈2.56 MeV as seen consistently in the simulations) using the presented method, would be expected to lase strongly in a state-of-the-art undulator with a period length of *λ*_u_=1.5 cm and undulator parameter *K*=1.0 to obtain FEL radiation at *λ*_r_≈0.32 nm. Because of the high-brightness electron beam source, the 1D FEL gain length may hence amount to *L*_g,1D_≈0.6 m, only, potentially allowing very high FEL power levels. With advanced cryogenic undulators which allow for sub-cm undulator periods^50^, hard X-ray wavelengths may be realizable already at even lower electron energies^8^. The control over the longitudinal phase space furthermore allows generation of beams with negative or positive chirps in a wide range, or even non-linear chirps by tuning the escort bunch current profile, which may be useful to realize chirped broadband radiation sources, energy tapering^51^ or (dechirped) multi-color light sources^52^. It should be noted that our findings, while they may on longer term prove transformative for future light source design and other applications which require extremely high beam quality, do not yet include extraction, capture, separation, transport and potential conditioning of the electron beams. This is beyond the scope of the present manuscript and a subject of future studies.

Finally, operation at long plasma wavelengths does not only allow a further reduction of the residual energy spread, but is also advantageous as regards experimental realization and shot-to-shot stability. If using an electron beam generated by a radiofrequency-based linear accelerator as driver for the plasma stage, the issue of synchronization of the electron beam with a laser pulse system arises. Inversely, this is also a problem which is found for external injection of linac-generated electron beams into laser-driven plasma waves^53^,^54^,^55^, and for pump-probe experiments and free-electron lasers^56^. In ref. 54, next to phase space rotation and compression in plasma waves, jitter effects are discussed, and in ref. 56, sub-30-fs synchronization has been reported, and improvements to sub-10-fs levels has been suggested. A longitudinal position jitter of ∼3 μm, corresponding to sub-10-fs temporal jitter, in a plasma wave with wavelength *λ*_p_ >300 μm amounts to less than 1%, and a dechirper region length of ∼10 μm suggests that the dechirping could be realized with each shot. In this connection, it suggests itself that the two release laser pulses which produce witness and escort bunches should be taken from the same laser system and parent laser pulse, such that they may inherently have fs or even sub-fs scale synchronization between each other (see Supplementary Fig. 3 and Supplementary Note 3). In all-optical versions of the scheme, in addition, the driver beam would also be produced by a laser system^8^,^10^,^12^,^57^,^58^,^59^,^60^,^61^,^62^, which would allow to harness inherently fs or sub-fs level synchronization. Spatial alignment jitter is in general of lesser concern than synchronization, because it has a reduced impact on trapping positions and hence residual energy spread. Furthermore, operation at larger plasma waves further improves resilience towards spatial positioning jitter, analogously to temporal jitter considerations. We conclude that state-of-the-art technology and methods of spatiotemporal jitter control are sufficient to realize the proposed approach.

## Methods

### Semi-analytical model of plasma wakes in the 1D nonlinear regime

A simplified 1D fluid model is used to describe the nonlinear wakefield excitation and interaction by the driver, witness and escort beams. The wake is driven by a relativistic electron beam with velocity *v*_d_ so that *v*_p_∼*v*_d_∼*c*, where *v*_p_ is the phase velocity of the plasma wake. In this relativistic, dephasing-free scenario in quasi-static approximation and assuming a non-evolving driver beam, the resulting quantities are only dependent on the longitudinal coordinate *ξ*=*z*−*v*_p_*t*. The Poisson equation can then be simplified^44^ to 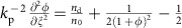, where *n*_0_ is the unperturbed background plasma density, *k*_p_=2π/*λ*_p_ is the plasma wavenumber corresponding to a plasma wavelength *λ*_p_=2π*c*[*m*_e_*ɛ*_0_/(*n*_0_*e*^2^)]^1/2^, *c* being the speed of light, *m*_e_ and *e* are the electron mass and charge, respectively, *n*_d_(*ξ)* is the electron driver charge density, and *φ* is the scaled electrostatic potential. If additional relativistically co-moving, non-evolving electron witness and escort beams with charge density distributions *n*_w_(*ξ)* and *n*_b_(*ξ)*, respectively, are added, the wake changes to 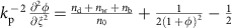. This differential equation is solved numerically (note that in Fig. 1, *n*_w_(*ξ)/n*_0_*=*0.2 to account for the much smaller witness bunch charge when compared to the escort), and expressions for the potential *φ* and the electric field *E*_*z*_ are obtained. The results shown in Fig. 1 are based on this description, and are depicted for lengths *σ*_*z*,d_=0.6/*k*_p,_ *σ*_*z*,w_=0.25/*k*_p_ and *σ*_*z*,b_=0.7/*k*_p_ and different ratios of *n*_b_*/n*_0_, which is used to inform the amount of charge to be released by the laser pulse generating the escort bunch in the implementation.

### 3D particle-in-cell simulations

Being guided by the semi-analytical 1D model, 3D particle-in-cell simulations using the VSim code^63^ have been carried through in order to verify the hypothesis. The driving electron beam is chosen as a Gaussian both in transverse (*x,y*) and longitudinal (*z*) direction, with r.m.s. sizes of *σ*_*x,y*_=7.5 μm and *σ*_*z*_=20 μm, respectively, a normalized emittance of *ɛ*_n_=5 mm-mrad, central energy of *W*_d_=10 GeV, energy spread Δ*W*_r.m.s._=0.1 GeV and total charge *Q*_d_=2 nC. Such energies and current levels are easily within the range of SLAC FACET-II. We chose a plasma density of *n*_0_=1.1 × 10^17^ cm^−3^, corresponding to a plasma wavelength of *λ*_p_≈100 μm. In view of the multi-cm-long simulation lengths, and the numerical Cherenkov effect, which is known to have significant ramifications on multi-dimensional relativistic plasma simulations, we use a co-moving simulation box with a grid of 288 × 48 × 48 cells (660 k cells in total), and a cell size of 0.8 μm in longitudinal and 3.2 μm in the transverse directions. We use the optimal time step^64^,^65^ and digital smoothing of the gridded currents to minimize the growth rate of numerical Cherenkov noise. Numerical Cherenkov is still significant and accounts for the wiggles in the lineouts of the electric field, as seen in Fig. 2—and for the production of an extra hump behind the escort beam, which leads to a substantially shorter usable length of the dechirping region than one would expect. The numerically usable dechirping length is ∼5 μm long and is adequate for demonstration of the concept; however, the longer physically correct dechirping length (estimated to be ∼10 μm) would allow significantly better results. We use an artificially short plasma wavelength to reduce the computational load, resulting in wakefield and driver field 'hot spots' of very high electric fields. This prohibits the use of H/He mixtures to realize the underdense photocathode PWFA because of He ionization at these hot spots, which impairs the laser-induced helium ionization and also leads to dark current^66^. Instead, we use a one-component version of the TH based on lithium, where Li is pre-ionized and the further ionization to Li^+^ is exploited to generate the laser-released bunches. This required setting the focus intensity of the Gaussian laser pulses, operating at a central wavelength of 0.8 μm, to much larger values than in the H/He case, namely to a normalized light amplitude of *a*_0_=*eE*/(*m*_e_*ωc*)≈0.1, where *e* and *m*_e_ are the charge and mass of an electron, respectively, *ω* is the laser frequency and *c* is the vacuum speed of light, which increases the emittance limits^67^,^68^. These choices also increase the longitudinal electric field gradient and hence the chirp of the produced beams. The validation of the scheme using a non-optimal physics scenario in order to reduce computational load leaves substantial room for further optimization of the performance.

### Residual energy spread scaling

The total energy spread of the generated electron bunches has a correlated energy spread component and an uncorrelated one. While the longitudinal phase space chirp which is imposed by the strong accelerating field gradient can be removed by the escort bunch technique, the uncorrelated energy spread is a result of the witness bunch generation process. This needs to be minimized, too, and can be estimated as follows. In doing so, one may assume a quasi-static ionization front position of the co-propagating laser pulse which releases the witness bunch. This is justified because of the stronger focusing, lower *a*_0_ and shorter laser pulse duration chosen when compared to the escort bunch. A typical effective ionization length for such laser pulses is of the order of 2*Z*_R_. Electrons which are released first are trapped first, and therefore experience a longer total acceleration time than electrons released later during the acceleration process. This constitutes a total residual maximum energy spread which can be expressed as Δ*W*_res,max_=*W*_first_−*W*_final_, where *W*_first_ is the energy gain of the first released witness electron and *W*_final_ is the energy gain of the last electron released. Figure 5a shows a color-coded longitudinal phase space of the electron witness bunch obtained from the simulation at the position of minimized total energy spread, where electrons born earlier are colored blue and electrons born later are colored red.

Since plasma wakefield acceleration driven by relativistic electron beams is essentially dephasing-free, the residual maximum energy spread can then be approximated as Δ*W*_res,max_=2 *E*_*z*,trap_ *Z*_R_, where *E*_*z*,trap_ is the accelerating longitudinal electric field at quasistatic trapping position of the witness bunch inside the wake. Taking into account the energy range of two full width at half maximum (FWHM) for the numerical evaluation, which contains approximately 99% of the contributing particles within 2 FWHM≈5*σ*_r.m.s._, allows to estimate the r.m.s. residual energy spread as Δ*W*_res,r.m.s._≈Δ*W*_res,max_/5≈2/5 *E*_*z*,trap_ *Z*_R_≈2π/5 *E*_*z*,trap_ *w*_0_^2^/*λ*_L_. Using the witness bunch laser parameters of *w*_0_=7 μm, *λ*_L_=800 nm, and an electric field at trapping position in the simulation of *E*_*z*,trap_≈33.2 GV m^−1^, this scaling predicts a Δ*W*_res,r.m.s._≈2.55 MeV, which is in excellent agreement with the minimum energy spread of Δ*W*_res,r.m.s._≈2.56 MeV, as retrieved from the simulation.

The residual energy spread scaling may be generalized by using the linear wave breaking limit *E*_0_ [V m^−1^]≅96 *n*_0_^1/2^ [cm^−3^] as estimation of the electric accelerating field, where *n*_0_ is the background plasma density in cm^−3^. While the peak fields in the strongly nonlinear blowout case can exceed the linear wavebreaking prediction considerably, for reasons of stability and robustness of the acceleration process the trapping position in the nonlinear blowout should be chosen significantly further ahead within the blowout, where the accelerating fields are lower and are more linear. The linear wave breaking limit then is therefore a good approximation. The scaling then generalizes to Δ*W*_res,max_≈2π/5 *E*_*z*,trap_ *w*_0,w_^2^/*λ*_L_≈96 *n*_0_^1/2^ [cm^−3^] 2π/5 *w*_0,w_^2^/*λ*_L_.

### Data availability

Data associated with research published in this paper is available at http://dx.doi.org/10.15129/3563d476-7a65-497c-9c7e-5a1f7a57591f.

## Additional information

**How to cite this article:** Manahan, G. G. *et al*. Single-stage plasma-based correlated energy spread compensation for ultrahigh 6D brightness electron beams. *Nat. Commun.* **8**, 15705 doi: 10.1038/ncomms15705 (2017).

**Publisher's note:** Springer Nature remains neutral with regard to jurisdictional claims in published maps and institutional affiliations.

## Supplementary Material

Supplementary Information Supplementary Figures, Supplementary Table and Supplementary Notes.

Supplementary Movie 1 Ultrahigh 6D brightness electron beam generation.
