# Supplementary material for: Single-stage plasma-based correlated energy spread compensation for ultrahigh 6D brightness electron beams
Source: Nat Commun. 2017 Jun 5;8:15705. doi: 10.1038/ncomms15705 (PMC5465367; doi:10.1038/ncomms15705)
Supplement: Supplementary Information — Supplementary Figures, Supplementary Table and Supplementary Notes. [file ncomms15705-s1.pdf]

Supplementary Figures

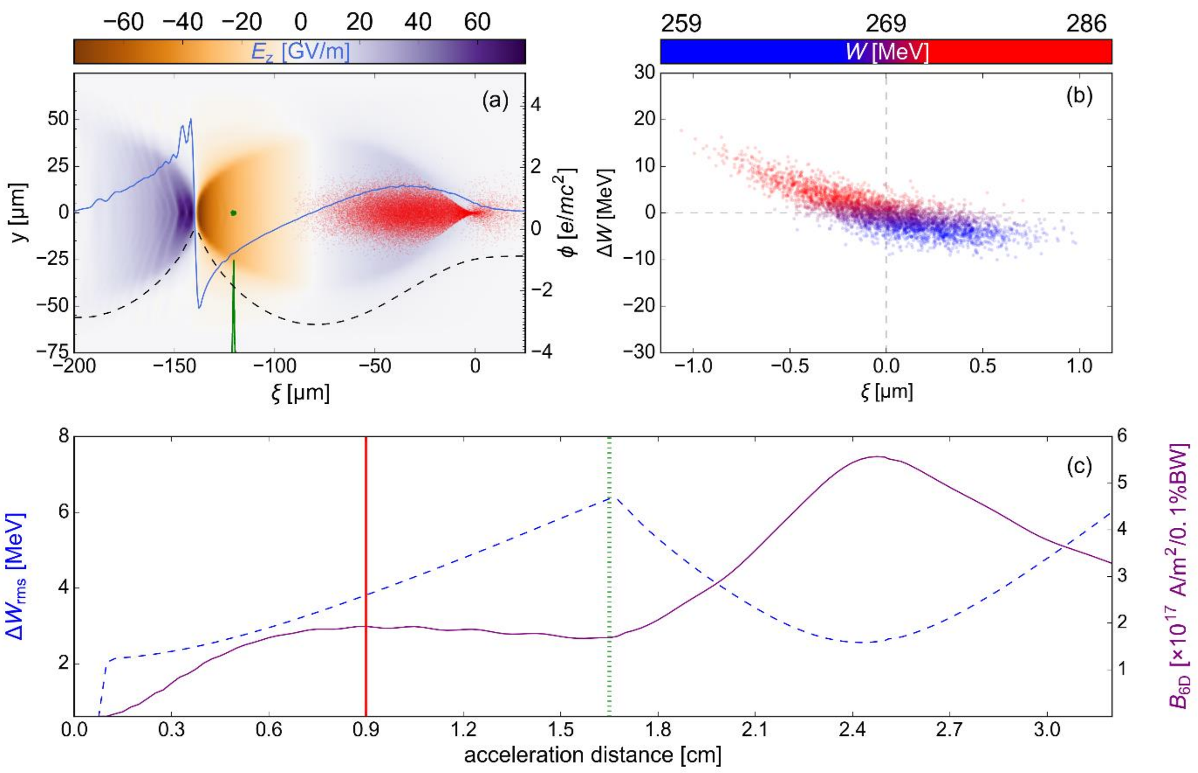

Supplementary Figure 1: Simulation of the dechirping process. Single snapshot of the supporting movie at z_{acc} =1.5 cm. The top left panel (a) shows the general features of the plasma wave, including drive beam and injected charge, the top right panel (b) shows the longitudinal phase space of the witness beam, and the bottom plot shows the witness bunch energy spread and 6D-brightness evolution.

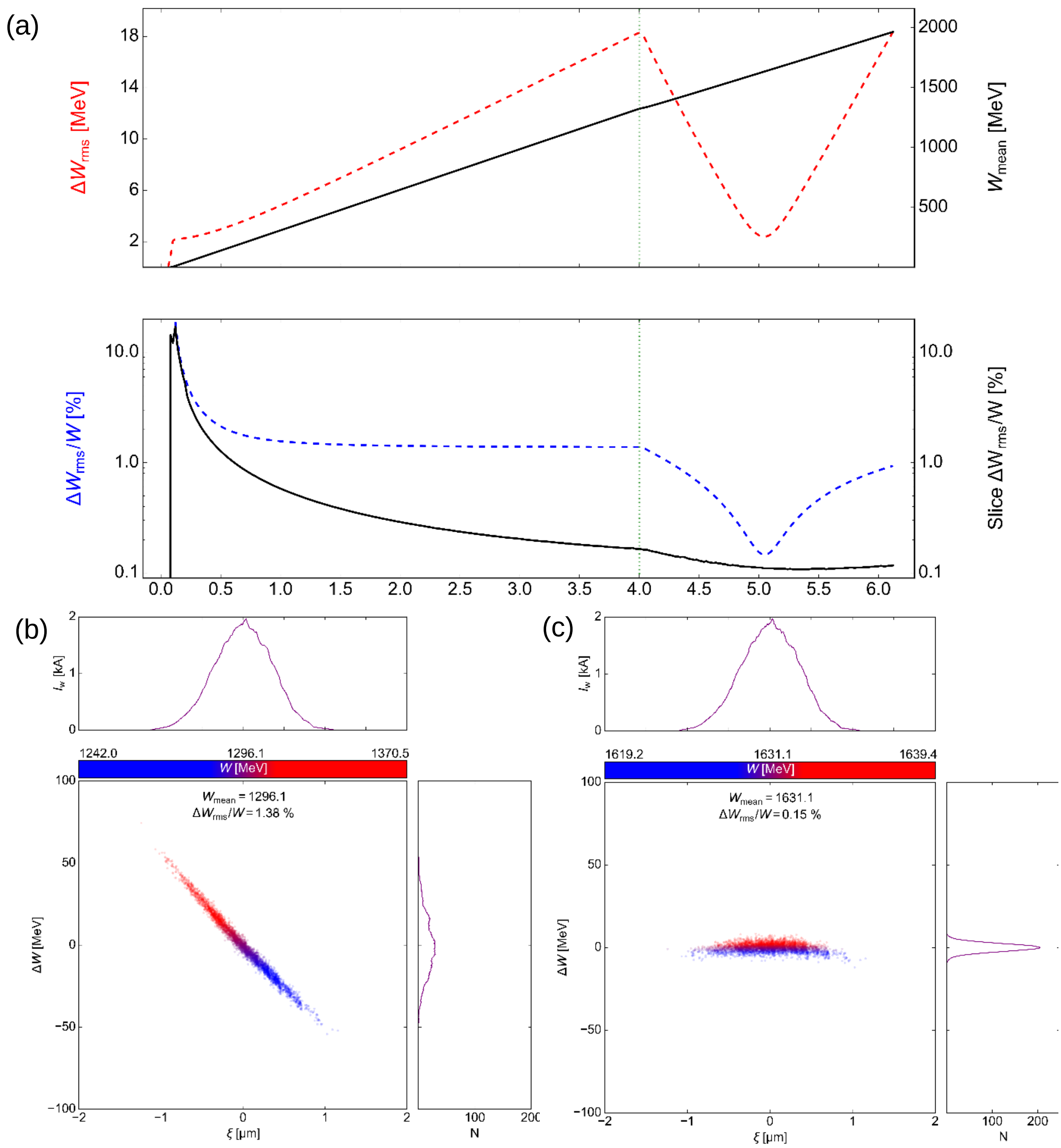

Supplementary Figure 2: Witness beam energy and energy spread evolution. Energy and energy spread evolution (a) of the injected witness beam when accelerated and dechirped at GeV+ energies. The green dashed line indicates the location when the escort beam is released. The longitudinal phase space of the witness beam just before the escort beam is released (b) at z_{acc}= 4.0 cm, and at minimum energy spread after chirp compensation (c).

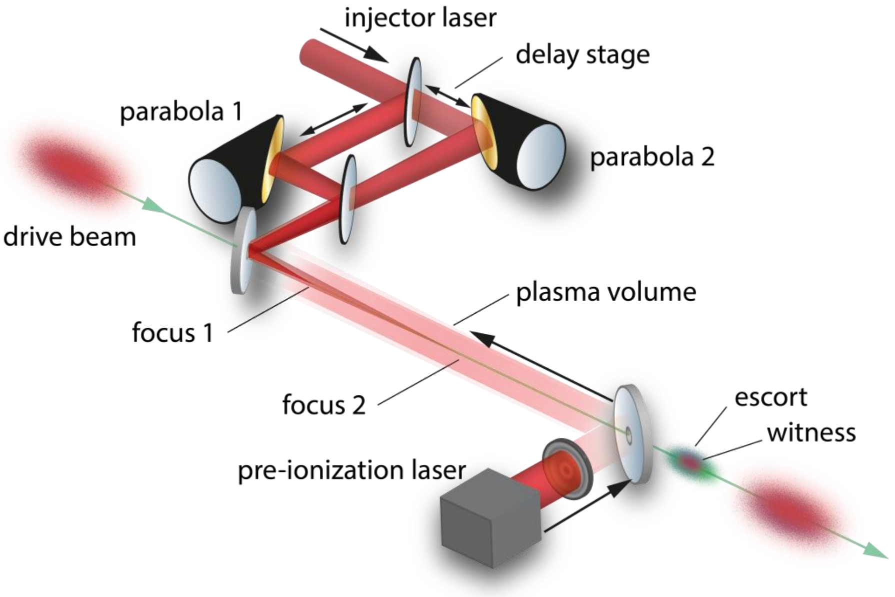

Supplementary Figure 3: Potential experimental setup for the dechirping technique. A plasma volume is generated through laser pre-ionization, while a high-current electron beam (red) drives the nonlinear plasma waves. Two off-axis parabolic mirrors with different focal lengths are set up with a delay stage and individual pulse compression to locally adjust the focal positions independent from the driver beam. Focus 1 releases the witness bunch (purple), while focus 2 releases the escort bunch (green) to initialize the energy spread compensation.

Supplementary Table 1: **Beam parameters of the driving electron beam.** Summary of the beam parameters (similar to a FACET-II electron beam) used to drive a plasma wave with wavelength λ_p = 100 µm.

Mean energy, W_d	10 GeV
Energy spread, ΔW_{rms}	0.1 GeV
Charge, Q_d	2 nC
Transverse size, $\sigma_{x,y}$	7.5 µm
Bunch length, σ_z	20 µm
Norm. emittance, ε_{rms}	5 mm-mrad

Supplementary Notes

Supplementary Note 1: Description of the 3D-PIC simulation movie visualizing generation of ultrahigh 6D-brightness electron bunches

The Supplementary Movie visualizes the generation of the ultrahigh 6D-brightness electron beams in the co-moving window mode, based on the 3D particle-in-cell code VSim. The electron beam parameters are summarized in Supplementary Table 1.

A single snapshot of the simulation movie is shown in Supplementary Figure 1 after an acceleration distance of z_{acc} = 1.5 cm, just before the escort electron beam generation. The top left panel (a) shows the geometry of the electron beam-driven plasma wave in a 2D projection of the longitudinal electric field E_z (purple is decelerating, orange is accelerating) and its on-axis lineout (blue), trailing the driver beam (red). Also shown are the laser-injected electron population (green) with the corresponding longitudinal normalized charge density profile (green solid line) and a lineout of the on-axis electrostatic potential ϕ (black dashed line). The right top panel (b) shows the witness electron bunch color-coded longitudinal phase space evolution, where the energy distribution ΔW is plotted as a function of the co-moving coordinate $\xi = z - ct$. At a mean energy of $W \approx$ 470 MeV, the witness electron beam has developed a substantial negative energy chirp and the longitudinal phase space rotates clockwise. Panel (c) shows the development of the witness bunch rms energy spread ΔW_{rms} (blue dashed line) and the 6D-brightness B_{6D} (purple solid line) during the acceleration. The vertical red solid line moves from left to right and indicates the momentary position within the acceleration process, while the vertical static green dashed line marks the location of the escort bunch production in the laboratory frame. The dechirping process is initiated when the escort bunch is released at $z_{acc} \approx$ 1.6 cm and overlaps with the witness bunch, resulting in the counter-clockwise rotation of the longitudinal phase space. This leads to the desired reduction of the energy spread and consequently to the increase of 6D-brightness.

Supplementary Note 2: Numerical study of energy spread compensation for higher energy witness electron energy

A 3D PIC VSim simulation has been performed at a reduced spatial grid resolution to be able to demonstrate the applicability of the technique to higher witness bunch energies. Here, the witness bunch is accelerated to ~1.2 GeV energy before the release of the escort bunch. This scenario is realized with a longitudinal grid size of 1.5 µm (instead of 0.8 µm as in the simulation discussed in the main manuscript and shown in the movie described in Supplementary Note 1) to be able to realise multi-cm acceleration lengths with the available computational resources.

Analogously to Figure 4a in the main manuscript, the top panel of Supplementary Figure 2a shows the energy gain of the witness electron bunch (solid black line, right *y*-axis) and the corresponding absolute energy spread (dashed red line, left *y*-axis). The production of the escort bunch is indicated by the dashed green line and here sets in after an acceleration distance of $z_{acc} \approx$ 4.0 cm, when compared to $z_{acc} \approx$ 1.65 cm as in the simulation underlying Figure 4 of the main manuscript. Consequently, the absolute energy spread increases approximately linearly to much higher values of $\Delta W_{rms} \approx$ 18 MeV when compared to $\Delta W_{rms} \approx$

6.5 MeV (see Figure 4a). However, the release of the escort bunch effectively cancels the absolute energy spread until the residual energy spread level of about $\Delta W_{\mathrm{res,rms}} \approx 2.6$ MeV – very similar to the lower energy case example – is reached due to longitudinal phase space rotation, facilitated by the escort bunch. Naturally, the relative energy spread at these higher energies is even smaller than in the lower energy example. The bottom panel of Supplementary Figure 2a shows the relative energy spread $\Delta W_{\mathrm{rms}}/W$ in percent (blue dashed line, left logarithmic y-axis), which reaches a minimum value of $\Delta W_{\mathrm{rms}}/W \approx 0.15$ % (at mean energy of $W \approx 1.63$ GeV), and the corresponding slice energy spread (black solid line, right logarithmic y-axis), which reduces down to nearly 0.1 %. The relative and slice energy spread values are quite similar, which is a signature of the efficiency of the dechirping method, leaving the residual energy spread as the dominating contribution.

Supplementary Figure 2b and 2c provides the longitudinal phase space of the witness bunch just before (b) release of the escort bunch, and at position of minimum energy spread in (c). The vertical projection of the longitudinal phase space shows that the kA-level witness current I is unaffected by the energy spread compensation process, and the horizontal projection shows the dramatic relative energy spread reduction from $\Delta W_{\mathrm{rms}}/W \approx 1.38$ % to $\Delta W_{\mathrm{rms}}/W \approx 0.15$ %. The color coding further visualizes the chirp (Supplementary Figure 2b) and residual energy spread (Supplementary Figure 2c).

Supplementary Note 3: Potential experimental setup configuration

In Supplementary Figure 3, a potential experimental setup is sketched. The drive beam propagates from top left to bottom right and thus defines the axis of acceleration. For PWFA, unless the driver is strong enough to self-ionize a low ionization threshold component of the gas medium, has to propagate in selectively pre-ionized medium. This can be done, for example, by a pre-ionization laser pulse which may be focused by an axicon or axilens as in recent campaigns at FACET to keep the spatial footprint small and to generate a wide plasma channel. The pre-ionization laser is shown in counter-propagating geometry here. Two synchronized release laser pulses, preferably originating from one and the same laser system in order to increase the stability, are then required to generate the witness at focus position 1 and the escort at focus position 2 to initiate the correlated energy chirp compensation. In this configuration, two off-axis parabolic mirrors, beamsplitters, a delay stage and an on-axis holed turning mirror are shown, thus realizing the required independent energy and delay tunability of both release laser pulses. The pre-ionization laser and plasma profile, and the relative focus position of both release lasers then defines the acceleration and dechirping length, before the electron beams exit the pre-ionized plasma region.
